# Pathological Manifestations of *Francisella orientalis* in the Green Texas Cichlid (*Herichthys cyanoguttatus*)

**DOI:** 10.3390/ani11082284

**Published:** 2021-08-03

**Authors:** Chia-Hsuan Chang, Sayuj Poudyal, Theeraporn Pulpipat, Pei-Chi Wang, Shih-Chu Chen

**Affiliations:** 1 Department of Veterinary Medicine, College of Veterinary Medicine, National Pingtung University of Science and Technology, Pingtung 91201, Taiwan; sharonkobeii@gmail.com; 2 International Degree Program of Ornamental Fish Technology and Aquatic Animal Health, International College, National Pingtung University of Science and Technology, Pingtung 91201, Taiwan; sayuz.pdl@gmail.com; 3 Department of Farm Resources and Production Medicine, Faculty of Veterinary Medicine, Kasetsart University Kamphaeng Saen Campus, Nakhon Pathom 73140, Thailand; pidpudpan@gmail.com; 4 Southern Taiwan Fish Diseases Research Center, College of Veterinary Medicine, National Pingtung University of Science and Technology, Pingtung 91201, Taiwan; 5 Research Center for Fish Vaccine and Diseases, College of Veterinary Medicine, National Pingtung University of Science and Technology, Pingtung 91201, Taiwan; 6 Research Center for Animal Biologics, National Pingtung University of Science and Technology, Pingtung 91201, Taiwan

**Keywords:** Cichlidae, green Texas cichlid, *Francisella*, pathogenicity

## Abstract

**Simple Summary:**

The following study demonstrates the pathological manifestations of an emerging virulent bacterium, *Francisella orientalis,* in an ornamental cichlid fish, the green Texas cichlid (*Herichthys cyanoguttatus*). This study was conducted to prove that *Francisella orientalis* can cause a disease in the green Texas cichlid that is similar to natural infection. *Francisella orientalis* was discovered for the first time in green Texas cichlid by our team in Taiwan in 2015. The present study simply tried to prove the susceptibility of *Francisella orientalis* in green Texas cichlid by conducting a challenge experiment, where healthy fish were injected with a dose of the bacteria. After the challenge, the healthy fish showed the same disease progression as was seen in the case of natural outbreak. The mortality rate, clinical symptoms, gross findings, and histopathological findings were similar to natural infection. *Francisella orientalis* could also be recovered in artificial media from challenged fish, thus indicating that the bacteria had multiplied inside the fish. These findings prove that green Texas cichlid (*Herichthys cyanoguttatus*) is susceptible to *Francisela orientalis,* and new management and vaccination strategies are necessary in the farming of this fish. This study also helps by adding to the knowledge of the growing host base for *Francisella orientalis***.**

**Abstract:**

*Francisella orientalis* (*Fo*) is considered to be one of the major pathogens of tilapia because of the high mortalities observed during outbreaks. Other cichlids belonging to the same family (Cichlidae) as tilapia are also quite susceptible to this pathogen. On various occasions, *Fo* has also been isolated from other warm water fish, including three-line grunt, hybrid striped bass, French grunt, Caesar grunt, and Indo-Pacific reef fish. However, only a few studies have reported the pathogenicity of *Francisella orientalis* in ornamental cichlid fish. This study fulfills Koch’s postulates by showing that a strain of *Fo* obtained from green Texas cichlid (*Herichthys cyanoguttatus*) was able to produce the same pathogenicity in healthy fish. A mortality of 100% was observed after healthy green Texas cichlid were experimentally injected with *Fo* at a dose of 8.95 × 10^5^ CFU/fish. DNA extracted from the organs of predilection (spleen, head kidney) gave positive results by PCR for all fish that died during the experimental period. Spleen and head kidney presented with multifocal white nodules in the affected fish, corresponding to typical vacuolated granulomas on histopathological examination of the tissues. Based on the results of this study, it is evident that *Fo* can indeed infect green Texas cichlid and produce a disease typical of francisellosis.

## 1. Introduction

Bacteria belonging to the genus *Francisella* are Gram negative, non-motile, strictly aerobic, facultatively intracellular, and pleomorphic coccobacilli. Fish-pathogenic *Francisella* are emerging pathogens that affect a wide range of aquatic animal hosts [1,2]. *Francisella noatunensis* (*Fn*) affects cold-water fish, including Atlantic salmon and Atlantic cod [3,4], and *Francisella orientalis* (*Fo*) affects warm-water fish such as tilapia [5,6,7,8,9,10,11,12,13,14,15,16,17,18,19], ornamental cichlids [18,20,21], three-line grunt [22], hybrid striped bass [23], French grunt and Caesar grunt [24], and Indo-Pacific reef fish [25]. However, this pathogen is most prevalent in tilapia and has only occasionally been seen in other hosts. Even though the bacteria was observed in tilapia in 1994 [5], it was only referred to as a Ricketssia-like organism, due to limitations in diagnosis. It was only years later confirmed as *Francisella,* after genetic diagnostic techniques were also developed. The difficulty in the identification of this bacteria in the first few years after its discovery was also due to its fastidious nature; they are difficult to grow in conventional media and require specialized media for their growth [15]. The disease is characterized by a systemic granulomatous infection, with clinical symptoms such as skin ulcers and multiple white nodules in visceral organs, including the spleen, head kidney, liver, gills, intestine, and heart [2]. The nodules are most extensively found in the spleen and head kidneys. These white nodules correspond to loosely-spaced vacuolated granulomas on histopathological examination [26]. *Fo* causes massive mortalities in tilapia and have been reported to be up to 95% [5,6]. Regarding the occasional outbreaks seen in other warm-water fish, one of the most common is in ornamental cichlids that belong to the same family (Cichlidae) as tilapia. Over the years, this pathogen has been detected in ornamental cichlids such as African Malawi cichlids and neon jewel cichlids (*Hemichromis bimaculatus*) [18,20,21,27]. In 2015, a strain of *Fo* was isolated from green Texas cichlid (*Herichthys cyanoguttatus*) in southern Taiwan [18]. This was the first case of *Fo* in this fish species. However, the detailed pathological manifestation of *Fo* in this species has not been studied. Thus, the present study was conducted to confirm susceptibility and to observe the pathogenicity of *Fo* in green Texas cichlid. The findings from this study will help to establish that a new fish species is susceptible to the emerging *Fo.* This study was conducted to satisfy the following objectives, and hence fulfill Koch’s postulates:

- To recover *Fo* bacteria from a diseased fish displaying symptoms typical of francisellosis.
- To isolate the bacteria from the diseased fish and grow it in pure culture.
- To challenge healthy fish with the bacteria in order to replicate the disease in the healthy fish.
- To reisolate the bacteria from the challenged fish and confirm it as being identical to the original bacteria.

## 2. Materials and Methods

### 2.1. Bacterial Strain and Growth Conditions

*Francisella orientalis* AOD104005 was isolated from a natural outbreak of disease in green Texas cichlid in Pingtung, Taiwan in 2015 by our team [18]. The infected fish showed splenomegaly and renomegaly, and multifocal white nodules were found throughout the spleen and kidney tissues. Bacteria were recovered from the infected fish in cysteine heart agar with 2% bovine hemoglobin solution (CHAB) after 60 h of incubation at 25 °C.

### 2.2. Fish for Experiment and Challenge

Twenty eight healthy green Texas cichlids were divided into two groups: a control group and an experimental group, with 14 fish in each group. The fish were kept in 250 L recirculatory tanks and the water temperature was maintained at 25 ± 2 °C. For the preparation of bacterial suspension, *Francisella orientalis* AOD104005 was grown in CHAB and incubated for 60 h at 25 °C. Pure colonies were suspended in phosphate buffered saline (PBS) (pH 7.4) and adjusted to an absorbance of OD_600_ = 1. Serial dilutions were made and the drop plate method was used to retrospectively determine the CFU/mL concentration. A dose of 8.95 × 10^6^ CFU/mL suspension was prepared and each fish in the experimental group was intraperitoneally injected with 0.1 mL of the suspension, amounting to a dose of 8.95 × 10^5^ CFU/fish. The control groups were similarly injected with 0.1 mL of PBS. The fish were observed daily for any clinical symptoms and the cumulative mortality was recorded up to 21 days.

### 2.3. DNA Extraction and PCR

Organs of predilection (spleen, head kidney) were collected from the dead fish for DNA extraction. Reagents from Genomaker were used for the extraction, combined with the phenol-chloroform phase separation technique [28]. Briefly, the organs were suspended in Genomaker reagent and macerated using glass beads. Phenol:chloroform (1:1) solution was added and centrifuged at 12,000 rpm for 5 min. The supernatant was separated and isopropanol was added followed by centrifugation at 12,000 rpm for 5 min for the precipitation of nucleic acids. The precipitate was digested with RNAse solution to remove residual RNA and then washed by adding ethanol precipitate buffer and centrifuging at 12,000 rpm for 5 min. The precipitate was air dried and diluted in double distilled water (DDW). The concentration of DNA was measured by reading the absorbance at 240 nm and 260 nm in a nanospectrophotometer and was maintained to ~50 ng/dL.

PCR was performed with a Takara thermal cycler TP600 (Takara Bio Inc. Shiga-Ken, Japan) with the PCR components consisting of 2 μL dNTPs, 0.625 μL primers, 0.125 μL Taq polymerase, 2.5 μL buffer, and 5 μL of sample DNA, making up a volume of 25 μL by adding DDW. The cycling conditions were as follows: 94 °C for 5 min; 94 °C for 40 s, 55 °C for 40 s, 72 °C for 1 min (35 cycles); 72 °C for 10 min. *Francisella* genus- specific primers were used [29]:

Reverse: F5 5′-CCTTTTTGAGTTTCGCTCC-3′

Forward: F11 5′-TACCAGTTGGAAACGACTGT-3′

### 2.4. Histopathology

The organs showing gross lesions (spleen and head kidney) were preserved in 10% neutral buffered formalin for 48 h. After that, the organs were subjected to dehydration by passing the tissues through successive dilutions of alcohol, followed by clearing the alcohol in the tissues with xylene. The xylene in the tissues was replaced by hot paraffin wax at 60 °C and then cooled to solidify. Sectioning of the paraffin blocks was done at 5 μm thickness using a microtome, and the sections were placed on slides. The sections were then deparaffinized, cleared with xylene, dehydrated with alcohol, and finally stained with hematoxylin and eosin.

## 3. Results

### 3.1. Cumulative Mortality and Clinical Symptoms

**Figure 1:**
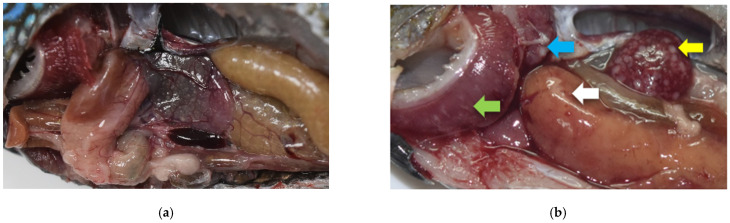
Gross pathological lesions of francisellosis observed in the challenged fish (**b**) when compared with the control fish (**a**) at day 11 post infection. Splenomegaly and renomegaly seen along with multifocal white modules in spleen (yellow arrow), head kidney (blue arrow), liver (white arrow), and gills (green arrow).

The fish in the experiment group showed decreased appetite for 3–4 days after the challenge. On the fifth day, they started developing clinical symptoms like lethargy, abnormal swimming posture, and sinking to the bottom of the tank. Fish began to die 7 days after the challenge in the experimental group and continued dying until the 12th day, at which point the mortality rate was 100%. No fish died in the control group, and they showed normal behavior throughout the experiment period. Upon necropsy of the dead fish (100%), gross lesions typical of francisellosis were observed throughout internal organs (Figure 1). Gills were pale, and white necrotic foci were observed (60%), followed by splenomegaly (100%), renomegaly (73%), and liver congestion (40%). In addition, multiple white nodules were also observed in the spleen (100%), kidney (73%), liver (87%), gills (73%), and heart (13%). Granuloma scores were given to each fish based on the number of nodules present in each organ (Table 1).

### 3.2. Organ Impression Smear and PCR

**Figure 2:**
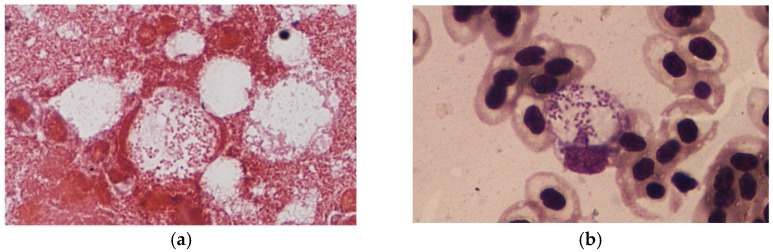
(**a**) Organ impression smear of spleen stained with Gram’s stain, showing Gram-negative coccobacilli inside macrophages; (**b**) blood impression smear stained with Liu’s stain (right) showing signet ring formation, with the bacteria displacing the nucleus of a macrophage to the periphery.

**Figure 3:**
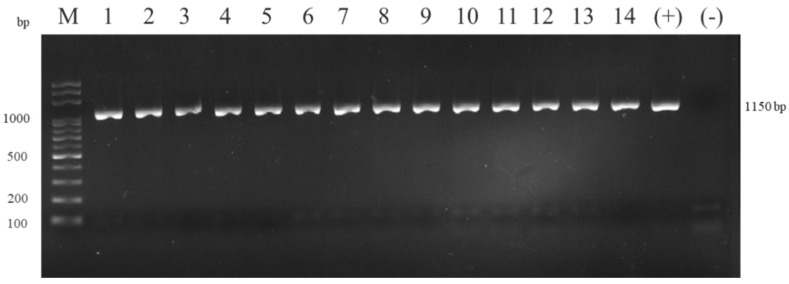
PCR assay of the 14 fish from the experimental group (Lanes 1–14). Pooled tissue samples (spleen and head kidney) from fish subjected to DNA extraction and PCR amplification yielding a product of 1150 bp using F11/F5 Francisella genus-specific primers after electrophoresis in 1.5% agarose gel. “M” = marker; “−” = negative control; “+” = positive control.

The spleen and kidney smears of the fish that died in the experimental group showed gram-negative coccobacilli, both intracellularly and extracellularly, by Gram staining and Liu’s staining (Figure 2a). Blood smears showed ring-like formations in macrophages, as the bacteria displaced the nucleus to the periphery of cells (Figure 2b). The DNA extracted from the lysate of spleen and head kidney of the fish that died in the experimental period were assessed by PCR and tested 100% positive for the presence of Francisella, using genus-specific primers yielding a product of 1150 bp after electrophoresing in 1.5% agarose gel (Figure 3).

### 3.3. Histopathological Examination

**Figure 4:**
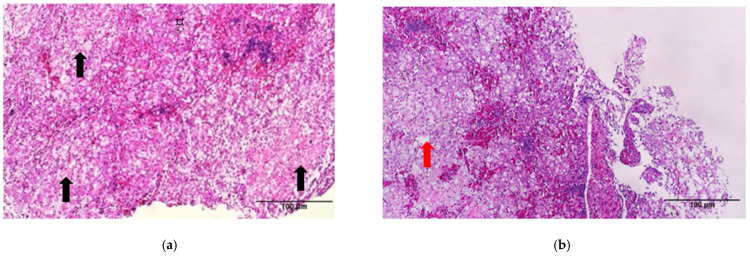

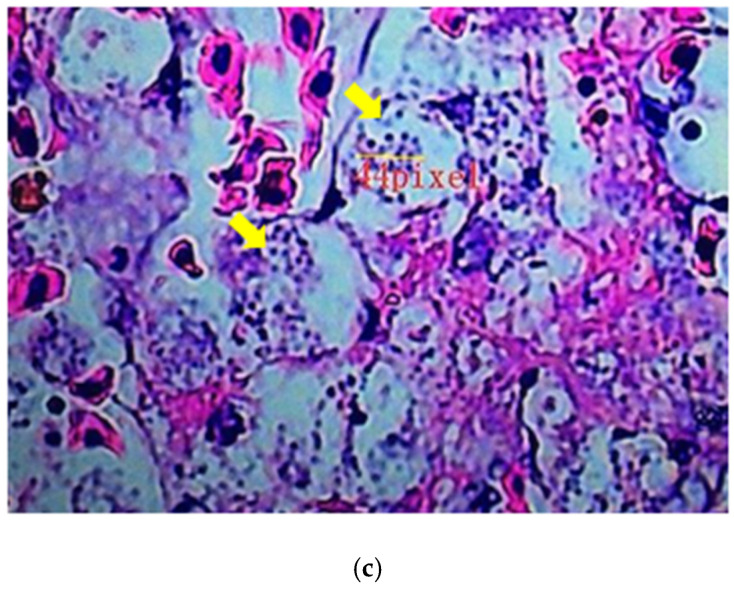
Histopathological sections of spleen tissues of dead fish from the experimental challenge group. (**a**) Spleen shows multifocal necrosis (black arrows). (**b**) A loosely formed, vacuolated granuloma typical of francisellosis observed in the spleen (red arrow). (**c**) At high magnification, bacterial cells are observed in the necrotic area inside vacuoles (yellow arrows) (H&E Stain).

**Figure 5:**
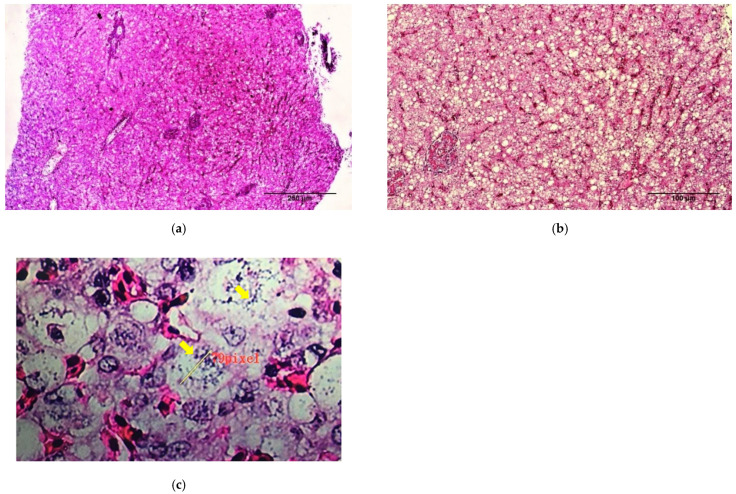
Histopathological sections of liver tissues of dead fish from the experimental challenge group. (**a**,**b**) Liver shows diffuse congestion (black arrows). (**c**) Vacuoles in the cytoplasm are filled with coccobacilli (yellow arrows) (H&E Stain).

**Figure 6:**
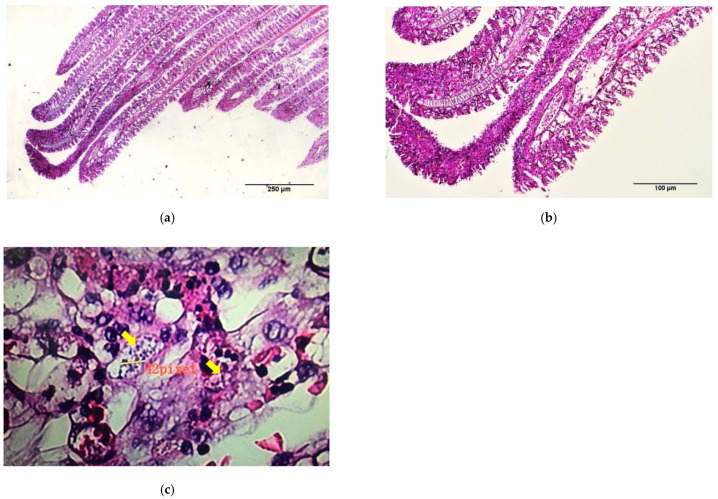
Histopathological sections of gill tissues of dead fish from the experimental challenge group. (**a**,**b**) The secondary gill lamella is infiltrated with inflammatory cells, showing the proliferation and fusion of the epithelial cells of the lamella (black arrows). (**c**) Typical coccobacilli can be seen inside the vacuoles (yellow arrows) (H&E Stain).

Histopathological examination of the dead fish revealed diffuse congestion and multifocal necrosis in the internal organs, including the spleen (Figure 4a) and liver (Figure 5a,b). The basal layer of the gill lamella showed proliferation, resulting in secondary gill lamella fusion and significant infiltration of inflammatory cells (Figure 6a,b). Loosely formed, vacuolated granulomas were seen in visceral organs, especially the spleen (Figure 4b). Macrophages infiltrated the parenchyma of different organs, particularly around the necrotic foci that formed granulomas, and the vacuolated macrophages were found to be harboring small coccobacilli (Figure 4c, Figure 5c, Figure 6c).

## 4. Discussion

*Francisella orientalis* (*Fo*) is an emerging, virulent bacterium causing high mortalities in tilapia and also infecting a variety of warm water fish, including ornamental cichlids, three-line grunt, hybrid striped bass, French grunt, Caesar grunt, and Indo-Pacific reef fish. The present study was conducted to confirm the pathogenicity of a strain of *Fo* in the ornamental cichlid, green Texas cichlid (*Herichthys cyanoguttatus*), obtained from an outbreak of francisellosis in the same fish in Pingtung, Taiwan in 2015. This study fulfills Koch’s postulates, hence determining a causative relationship between the microbe and the disease [30] and confirming that *Fo* is pathogenic to this species of fish. In fact, owing to the fact that the experimental group showed a mortality of 100%, it can be assumed that *Fo* is highly pathogenic to the species. This finding warrants new management and vaccination strategies, and which need to be employed in the farming of this cichlid species.

In this study, a gradual mortality pattern was observed in the experimental group with the fish starting to die from day 7 post infection and all of the fish dying by the 12th day when injected with a dose of 8.95 × 10^5^ CFU/fish. This mortality pattern is similar to the mortality observed in tilapia when challenged with the same dose of another strain of *Fo* [18]. However, when challenged with the same dose, tilapia started dying from day 3 post infection, while in this experiment, green Texas cichid started dying from day 7 post infection. This difference could be because of the difference in strain or because the pathogenesis of *Fo* may vary between species; as was the case in one of our previous experiments involving Asian seabass and largemouth bass, where the onset of mortality was different compared to tilapia, even when the same strain was used to challenge all the fish [28]. The gross pathological signs, including splenomegaly and renomegaly, along with multifocal white nodules in the spleen, kidney, gills, liver, heart, and intestine are also similar to the pathological manifestations observed in tilapia [28]. This shows that *Fo* is pathogenic to the two species. However, cases of natural outbreak of francisellosis in cichlids are rare compared to tilapia. In fact, to our knowledge, this is the only case of francisellosis seen in green Texas cichlid. Other experiments assessing different doses of bacteria must also be conducted on the green Texas cichlid, where different doses could be used to create a mortality profile and also to determine the median lethal dose. Further experiments should focus on infecting the green Texas cichlid with different strains of *Fo* isolated from other cichlids and assessing the differences in pathogenicity with the different strains. Further experiments can also concentrate on determining whether fish can be infected with other routes of infection such as oral, immersion, or cohabitation, which closely resemble the natural modes of infection. The reason why fewer natural cases of francisellosis outbreak are seen in ornamental cichlids when compared to tilapia can also be simply because tilapia farming is more extensive compared to ornamental cichlid farming. Today, the total global tilapia production has increased to nearly 7 million tonnes per year [31], with only a fraction of that being ornamental cichlid production. Regardless, more pathogenicity studies are needed to accurately assess the pathogenic potential of this bacteria in the green Texas cichlid.

Bacteria belonging to the genus *Francisella* are intracellular in nature and have been found to infect macrophages, dendritic cells, polymorphonuclear neutrophils, hepatocytes, endothelial, and type II alveolar lung epithelial cells for survival and proliferation [32,33,34,35]. In the present study, impression smears showed bacteria residing within the macrophages of spleen and kidney tissues and also in the blood. This is explanatory since the bacteria has also been found to have an extracellular phase during its life cycle [35,36]. Bacteria were also seen to change the structure of macrophages to signet ring cells when the bacteria multiplying inside a cell pushed the nucleus of the cell to the periphery (Figure 2b). This phenomenon was also observed in previous studies of francisellosis in tilapia [5,6,18]. *Francisella* are seen to multiply and proliferate in macrophages and other phagosomes before rupturing the cell and escaping into the cytosol, where they are promptly engulfed by other phagosomes [35]. The histopathological manifestations were similar to the natural infections of francisellosis typical of loosely spaced vacuolated granulomas in the visceral organs. Furthermore, multiple localized cell degeneration was observed, along with necrosis and congestion in the spleen and liver. All these signs are typical of francisellosis in fish [1,2,5].

## 5. Conclusions

The present study was conducted with the objective to confirm the susceptibility of green Texas cichlid to francisellosis by fulfilling Koch’s postulates. The *Fo* bacteria that were recovered from a natural outbreak of francisellosis in an ornamental cichlid farm in Pingtung, Taiwan were isolated in pure culture and challenged to healthy green Texas cichlid, after which the fish displayed clinical signs similar to the natural infection. This was confirmed by gross and histopathological findings. The bacteria was then reisolated from the challenged fish and was confirmed to be the same bacteria as in the outbreak, by using bacteriological tests and PCR confirmation. Thus all of Koch’s postulates were fulfilled, confirming the susceptibility of green Texas cichlid to francisellosis.

## Figures and Tables

**Table 1:** Necropsy results of *Fo* infected fish that died in the experimental period.

Degree of Severity	Gills	Spleen	Kidney	Liver	Heart	Intestine
−	4	0	4	2	13	15
+	6	2	1	7	1	0
++	5	0	3	1	1	0
+++	0	13	7	5	0	0

“−” = no nodules, “+” = 1–5 nodules per organ, “++” = 6–10 nodules per organ, “+++” = more than 10 nodules per organ.

## Data Availability

All data generated or analyzed during this study are included in this published article.

## References

[1] Birkbeck T.H., Feist S.W., Verner-Jeffreys D.W. (2011). *Francisella* infections in fish and shellfish. J. Fish Dis..

[2] Colquhoun D.J., Duodu S. (2011). *Francisella* infections in farmed and wild aquatic organisms. Vet. Res..

[3] Birkbeck T.H., Bordevik M., Frøystad M.K., Baklien Å. (2007). Identification of *Francisella* sp. from Atlantic salmon, *Salmo salar* L., in Chile. J. Fish Dis..

[4] Ellingsen T., Inami M., Gjessing M.C., Van Nieuwenhove K., Larsen R., Seppola M., Lund V., Schrøder M.B. (2011). Francisella noatunensis in Atlantic cod (Gadus morhua L.); waterborne transmission and immune responses. Fish Shellfish Immunol..

[5] Chen S.C., Tung M.C., Chen S.P., Tsai J.F., Wang P.C., Chen R.S., Lin S.C., Adams A. (1994). Systematic granulomas caused by a rickettsia-like organism in Nile tilapia, *Oreochromis niloticus* (L.), from southern Taiwan. J. Fish Dis..

[6] Chern R.S., Chao C.B. (1994). Outbreaks of a disease caused by rickettsia-like organism in cultured tilapias in Taiwan. Fish Pathol..

[7] Mauel M.J., Soto E., Moralis J.A., Hawke J. (2007). A piscirickettsiosis-like syndrome in cultured Nile tilapia in Latin America with *Francisella* spp. as the pathogenic agent. J. Aquat. Anim. Health.

[8] Soto E., Hawke J.P., Fernandez D., Morales J.A. (2009). *Francisella* sp., an emerging pathogen of tilapia, *Oreochromis niloticus* (L.), in Costa Rica. J. Fish Dis..

[9] Ottem K.F., Nylund A., Karlsbakk E., Friis-Moller A., Kamaishi T. (2009). Elevation of *Francisella philomiragia* subsp. *noatunensis* to *Francisella noatunensis* comb. nov. [syn. *F. piscicida* Ottem et al. (2008) syn. nov.] and characterization of *F. noatunensis* subsp. *orientalis* subsp. nov., two important fish pathogens. J. Appl. Microbiol..

[10] Jeffery K.R., Stone D., Feist S.W., Verner-Jeffreys D.W. (2010). An outbreak of disease caused by *Francisella* sp. in Nile tilapia *Oreochromis niloticus* at a recirculation fish farm in the UK. Dis. Aquat. Org..

[11] Iregui C., Vasquez G.M., Rey A., Verjan N. (2011). Piscirickettsia-like organisms as a cause of acute necrotic lesions in Colombian tilapia larvae. J. Vet. Diagn. Investig..

[12] Lin Q., Li N., Fu X., Hu Q., Chang O., Liu L., Zhang D., Wang G., San G., Wu S. (2016). An outbreak of granulomatous inflammation associated with *Francisella noatunensis* subsp. *orientalis* in farmed tilapia (*Oreochromis niloticus* × *O. aureus*) in China. Chin. J. Oceanol. Limnol..

[13] Ortega C., Mancera G., Enríquez R., Vargas A., Martínez S., Fajardo R., Avendaño-Herrera R., Navarrete M.J., Romero A. (2016). First identification of *Francisella noatunensis* subsp. *orientalis* causing mortality in Mexican tilapia *Oreochromis* spp.. Dis. Aquat. Org..

[14] Jantrakajorn S., Wongtavatchai J. (2016). *Francisella* infection in cultured tilapia in Thailand and the inflammatory cytokine response. J. Aquat. Anim. Health.

[15] Nguyen V., Dong H., Senapin S., Pirarat N., Rodkhum C. (2015). *Francisella noatunensis* subsp. *orientalis*, an emerging bacterial pathogen affecting cultured red tilapia (*Oreochromis* sp.) in Thailand. Aquac. Res..

[16] Assis G.B.N., Tavares G.C., Pereira F.L., Figueiredo H.C.P., Leal C.A.G. (2017). Natural coinfection by *Streptococcus agalactiae* and *Francisella noatunensis* subsp. *orientalis* in farmed Nile tilapia (*Oreochromis niloticus* L.). J. Fish Dis..

[17] Hsieh C.Y., Tung M.C., Tu C., Chang C.D., Tsai S.S. (2006). Enzootics of visceral granulomas associated with *Francisella*-like organism infection in tilapia (*Oreochromis* spp.). Aquaculture.

[18] Pulpipat T., Lin K.H., Chen Y.H., Wang P.C., Chen S.C. (2019). Molecular characterization and pathogenicity of *Francisella noatunensis* subsp. *orientalis* isolated from cultured tilapia (*Oreochromis* sp.) in Taiwan. J. Fish Dis..

[19] Soto E., Shahin K., Talhami J.J., Griffin M.J., Adams A., Ramírez-Paredes J.G. (2019). Characterization of *Francisella noatunensis* subsp. *orientalis* isolated from Nile tilapia *Oreochromis niloticus* farmed in Lake Yojoa, Honduras. Dis. Aquat. Org..

[20] Hsieh C.Y., Wu Z.B., Tung M.C., Tsai S.S. (2007). PCR and in situ hybridization for the detection and localization of a new pathogen *Francisella*-like bacterium (FLB) in ornamental cichlids. Dis. Aquat. Org..

[21] Lewisch E., Dressler A., Menanteau-Ledouble S., Saleh M., El-Matbouli M. (2014). Francisellosis in ornamental African cichlids in Austria. Bull. Eur. Assoc. Fish Pathol..

[22] Kamaishi T., Fukuda Y., Nishiyama M., Kawakami H., Matsuyama T., Yoshinaga T., Oseko N. (2005). Identification and pathogenicity of intracellular *Francisella* bacterium in three-line grunt *Parapristipoma Trilineatum*. Fish Pathol..

[23] Ostland V.E., Stannard J.A., Creek J.J., Hedrick R.P., Ferguson H.W., Carlberg J.M., Westerman M.E. (2006). Aquatic *Francisella*-like bacterium associated with mortality of intensively cultured hybrid striped bass *Morone chrysops* × *M. saxatilis*. Dis. Aquat. Org..

[24] Soto E., Primus A.E., Pouder D.B., George R.H., Gerlach T.J., Cassle S.E., Johnson T., Boyd S., Handsel T., Yanong R.P.E. (2014). Identification of *Francisella noatunensis* in novel host species French grunt (*Haemulon flavolineatum*) and Caesar grunt (*Haemulon carbonarium*). BIOONE.

[25] Camus A.C., Dill J.A., McDermott A.J., Clauss T.M., Berliner A.L., Boylan S.M., Soto E. (2013). *Francisella noatunensis* subsp. *orientalis* infection in Indo-Pacific reef fish entering the United States through the ornamental fish trade. J. Fish Dis..

[26] Soto E., Wiles J., Elzer P., Macaluso K., Hawke J.P. (2011). Attenuated *Francisella asiatica* iglC mutant induces protective immunity to francisellosis in tilapia. Vaccine.

[27] López-Crespo R.A., Martínez-Chavarría L.C., Lugo-García A.T., Romero-Romero L.P., García-Márquez L.J., Reyes-Matute A. (2019). Outbreak of francisellosis (Francisella noatunensis subsp. orientalis) in cultured neon jewel cichlids Hemichromis bimaculatus from Morelos, Mexico. Dis. Aquat. Organ..

[28] Poudyal S., Pulpipat T., Wang P.C., Chen S.C. (2020). Comparison of the pathogenicity of *Francisella orientalis* in Nile tilapia (*Oreochromis niloticus*), Asian seabass (*Lates calcarifer*) and largemouth bass (*Micropterus salmoides*) through experimental intraperitoneal infection. J. Fish Dis..

[29] Forsman M., Sandström G., Sjöstedt A. (1994). Analysis of 16S Ribosomal DNA Sequences of *Francisella* Strains and utilization for determination of the phylogeny of the genus and for identification of strains by PCR. Int. J. Syst. Evol. Microbiol..

[30] Evans A.S. (1978). Causation and disease: A chronological journey: The Thomas Parran lecture. Am. J. Epidemiol..

[31] FAO (2020). Tilapia production and trade with a focus on India. World Aquaculture Performance Indicators (WAPI).

[32] Oyston P.C.F., Sjöstedt A., Titball R.W. (2004). Tularaemia: Bioterrorism defence renews interest in Francisella tularensis. Nat. Rev. Microbiol..

[33] Hall J.D., Craven R.R., Fuller J.R., Pickles R.J., Kawula T.H. (2007). Francisella tularensis replicates within alveolar type II epithelial cells in vitro and in vivo following inhalation. Infect. Immun..

[34] McCaffrey R.L., Allen L.-A.H. (2006). Francisella tularensis LVS evades killing by human neutrophils via inhibition of the respiratory burst and phagosome escape. J. Leukoc. Biol..

[35] Celli J., Zahrt T.C. (2013). Mechanisms of *Francisella tularensis* intracellular pathogenesis. Cold Spring Harb. Perspect. Med..

[36] Forestal C.A., Malik M., Catlett S.V., Savitt A.G., Benach J.L., Sellati T.J., Furie M.B. (2007). Francisella tularensis Has a Significant Extracellular Phase in Infected Mice. J. Infect. Dis..

